# Down Syndrome: Evaluating Disparities in Place of Death in the United States Using Centers for Disease Control and Prevention’s Wide-Ranging Online Data for Epidemiologic Research (CDC-WONDER) Database Over 22 Years

**DOI:** 10.7759/cureus.63212

**Published:** 2024-06-26

**Authors:** Deepanwita Biswas, Gauravi Shinde, Shishwa Mudiyala, Ximena Delgado, Arunika Korwar, Ayushi Rai

**Affiliations:** 1 Medicine, Bharati Vidyapeeth's Medical College, Pune, IND; 2 Internal Medicine, East European University, Tiblisi, GEO; 3 Pediatrics, Prathima Institute of Medical Sciences, Karimnagar, IND; 4 Internal Medicine, Cayetano Heredia University, Lima, PER; 5 Internal Medicine, KJ Somaiya Medical College, Mumbai, IND; 6 Internal Medicine, American University of Barbados, Wildey, BRB

**Keywords:** cdc-wonder database, end-of-life support, palliative care, hospice care, homecare, down syndrome, mortality trends

## Abstract

Introduction: The Uniform Determination of Death Act (UDDA) ensures that individuals with irreversible cessation of circulatory, respiratory, or brain functions receive timely palliative care. Our research has focused on identifying disparities in mortality among individuals with Down syndrome (DS) based on gender, age, racial groups, and geographic regions within the United States over 22 years. This study aims to analyze differences in the location of death, including hospitals, nursing homes, hospice care facilities, and unspecified locations, considering demographic and regional variables.

Methodology: Utilizing a cross-sectional observational study design, we extracted data from the Centers for Disease Control and Prevention's Wide-ranging Online Data for Epidemiologic Research (CDC-WONDER) database, specifically targeting deaths coded under the International Classification of Diseases, 11th Revision (ICD-11) code "Q-90." This analysis, covering 1999 to 2020, segmented the data by age, gender, race, and United States Census regions. Death locations were categorized into home/hospice, medical facilities, and nursing/other facilities. Data analysis was conducted using Microsoft Excel, and the Autoregressive Integrated Moving Average (ARIMA) model was applied for statistical assessments.

Results: Our analysis included 22604 deaths related to DS, as recorded in the CDC-WONDER database from 1999 to 2020. The majority of these deaths occurred in medical or nursing facilities, with home or hospice deaths accounting for 6106 cases and other locations for 5.29% of deaths. Univariate logistic regression was used to identify predictors of home or hospice deaths, revealing a trend of increasing deaths in these settings over time.

Conclusions: Between 1999 and 2020, there was a notable increase in the number of individuals with DS dying at home or in hospice care, especially among those aged 55-64. Female individuals and those identified as white experienced higher mortality rates than other demographic groups. This shift highlights the need to understand the disparity in places of death within this population, ensuring equitable access to quality end-of-life care for all individuals with DS.

## Introduction

“An individual who has sustained either (1) irreversible cessation of circulatory and respiratory functions or (2) irreversible cessation of all functions of the entire brain, including the brain stem, is dead,” as stipulated by the Uniform Determination of Death Act (UDDA) [1]. Death is an inescapable reality that all humans must eventually face. Nonetheless, it is imperative to take every possible measure to ensure that patients do not suffer pain. Providing palliative care and therapy promptly is essential to managing symptoms and prioritizing the comfort of the patient above all else. The duty falls on physicians, medical facilities, nursing staff, and families to ensure that patients receive this care. This can be achieved by providing patients with appropriate end-of-life care, which includes emotional, spiritual, and religious support; meeting their psychological needs; and ensuring they receive medical attention [2].

Trisomy 21, commonly known as Down syndrome (DS), is one of the most prevalent genetic disorders in the United States. It arises from an extra copy of chromosome 21, a consequence of sister chromatids failing to separate during cell division [3,4]. Women aged 35 years and older are at a higher risk of having a child with DS, and over the last 15 years, the prevalence of the disorder has substantially increased [5]. Children with DS are more susceptible to medical conditions, cognitive decline, and multi-organ comorbidities, potentially leading to premature mortality [5,6]. According to the National Survey of Children with Special Healthcare Needs (NSCSH), conducted between 2005 and 2006, a considerable number of families with children with DS have provided healthcare services at home. Specifically, nearly 60% of these families performed tasks such as changing bandages, managing feeding or breathing equipment, and administering medication and therapies [7]. Moreover, about 40% of families with children affected by DS had a family member who had to cease working due to the child’s condition. These findings underscore the vital role that family and home care play in supporting children with DS [7].

In contrast, a 2002 population-based study revealed that between 1983 and 1997, the median age of death for individuals with DS increased from 25 to 49 years [8]. The main factors contributing to decreased mortality rates among individuals with DS include routine follow-ups, physical examinations, laboratory tests, observation of growth patterns, behavior, and social skills; encouragement of individuality and independence; provision of opportunities for independent living, work, community service, and group home assistance when needed; and a reduction of exposure to specific environmental factors [5,6,8]. A critical component also involves ensuring that individuals with DS make an appropriate transition to adult medical care, enrolling them in hospice care; screening for symptoms; and addressing their pain, emotional, psychological, and spiritual needs [2,3,6].

According to 2015 research, despite the increased longevity of persons with DS in recent decades, African Americans and other minority groups have not experienced the same degree of improvement in mortality rates as their Caucasian counterparts. Life expectancy in minority populations has risen, though not to the same extent as in non-minority populations [9]. Furthermore, women with DS are more likely to die from dementia and Alzheimer’s disease compared to men with DS. Additionally, non-Hispanic whites are more likely to die from these conditions than non-Hispanic Blacks and Hispanics [10]. To explain this racial discrepancy, researchers have examined hundreds of factors, including prenatal care, preterm birth, and congenital heart disease [9]. Despite these advancements, there remains a critical need to comprehensively evaluate and understand disparities in DS mortality trends across different demographics. Consequently, the objective of this study was to assess the disparities in DS mortality trends among different sexes, ages, racial groups, and census regions in the United States over the past 22 years.

Aims and objectives

To evaluate disparities in the place of death in the United States, in (1) hospitals or medical facilities, (2) nursing homes, (3) homes, (4) hospice care, and (5) unknown settings for individuals with DS based on age, gender, race, and census region in the United States between 1999 and 2020.

## Materials and methods

In this retrospective study design, data was obtained from the Centers for Disease Control and Prevention (CDC) website, specifically through the Wide-ranging Online Data for Epidemiologic Research (WONDER) platforms. This database integrates data from the National Center for Health Statistics, encompassing comprehensive mortality statistics derived from the death certificates of individuals across the United States [11].

Data collection was conducted on October 16, 2023, focusing on the underlying causes of death over 22 years between 1999 and 2020, utilizing bridged-race categories. The selection criterion for identifying relevant cases was based on the ICD-11 code “Q-90” for DS [12,13]. Subsequently, the raw data was exported to Microsoft Excel, version 10, for further analysis.

To enhance the study’s comprehensiveness, the collected data were organized according to specific parameters, including age group, gender, census region, and race. Age grouping was conducted using a 10-year age range segmentation for both sexes. The racial demographic variables included American Indian or Alaska Native, Black or African American, White, and Asian or Pacific Islander. Geographical distinctions were made by classifying the data into four United States Census regions: Northeast, Midwest, South, and West. The data were also categorized by places of death, such as home or hospice (descendant’s home or hospice facility), medical or nursing facility (including inpatient, outpatient, ER, dead on arrival, unknown status, nursing home, or long-term care), and others.

The analysis focused on the total number of deaths for all years within the specified timeframe, categorizing the places of death into three distinct groups: (1) home or hospice, (2) medical facilities and nursing centers, and (3) others. To identify temporal patterns and trends, a statistical analysis was conducted using an Autoregressive Integrated Moving Average Model (ARIMA).

## Results

Data covering 22604 deaths related to DS between 1999 and 2020 were collected from the CDC WONDER database. The majority of deaths, accounting for 67.69% (n=15302), occurred in medical facilities or nursing centers, followed by 27.01% (n=6106) in homes or hospices. The smallest proportion, 5.29% (n = 1196), was reported in the “others” category. Additionally, predictors of deaths in homes or hospices were identified and analyzed using univariate logistic regression methods. Graphs depicting the data between 1999 and 2020 show a gradually increasing trend in these numbers.

Table 1 details the locations of deaths, noting occurrences in homes or hospices, medical facilities or nursing centers, and other settings across various age groups, genders, census regions, and races. Across all groups, the highest incidence of deaths (n=15302) occurred in medical facilities and nursing centers. Within these facilities, the largest number of deaths (n=6117) was observed in the 55-64 age group. Notably, the “others” category reported no deaths among the age groups of one to four years, five to 14 years, 75-84 years, and those over 85 years. Moreover, no deaths were recorded in the >85 years age group in homes and hospices.

**Table 1:** Number of deaths in different locations, grouped according to demographic variables The locations of patient deaths, specifying occurrences in home or hospice, medical facilities or nursing centers, and other places across various age groups, genders, census regions, and races. Data is presented as n (%).

Variables	Home or hospice (n=6106)	Medical facility or nursing (n=15302)	Others (n=1196)
10-year age groups			
< 1 year	90 (1.4%)	1700 (11.1%)	23 (1.92%)
1-4 years	52 (0.85%)	271 (1.77%)	0
5-14 years	44 (0.72%)	170 (1.11%)	0
15-24 years	148 (2.42%)	364 (2.37%)	12 (1%)
25-34 years	241 (3.94%)	526 (3.44%)	27 (2.25%)
35-44 years	409 (6.69%)	932 (6.09%)	44 (3.67%)
45-54 years	1727 (28.3%)	3440 (22.48%)	310 (25.9%)
55-64 years	2777 (45.48%)	6117 (39.98%)	603 (50.42%)
65-74 years	549 (8.99%)	1581 (10.33%)	141 (11.79%)
75-84 years	33 (0.54%)	124 (0.81%)	0
85+ years	0	38 (0.25%)	0
Gender			
Female	3099 (50.76%)	7314 (47.79%)	604 (50.5%)
Male	3007 (49.24%)	7988 (52.20%)	592 (49.5%)
Census region			
Census region 1: northeast	1074 (17.59%)	2886 (18.86%)	165 (13.8%)
Census region 2: midwest	1611 (26.38%)	4174 (27.28%)	358 (29.93%)
Census region 3: south	2244 (36.75%)	5100 (33.33%)	368 (30. 77%)
Census region 4: west	1177 (19.28%)	3142 (20.53%)	294 (24.58%)
Race			
American Indian or Alaska Native	35 (0.57%)	68 (0.44%)	0
Asian or Pacific Islander	64 (1.05%)	280 (1.829%)	17 (1.42%)
Black or African American	561 (9.19%)	1544 (10.1%)	81 (6.77%)
White	5436 (89.03%)	13399 (87.56%)	1091 (91.2%)

Gender-specific data showed that the highest number of deaths among males (n=7988) occurred in medical facilities and nursing centers and the lowest number of deaths (n=592) occurring in other categories.

In terms of census region classifications, the maximum number of deaths (n=5100) was reported in census region 3 (south) within medical and nursing facilities, whereas the minimum (n=165) occurred in census region 1 (northeast) within the “others” category.

With regard to race, the highest number of deaths (n=13399) was recorded among the White race, whereas there were no deaths in the “others” category for American Indian or Alaska Native individuals.

Table 2 outlines the predictors of home and hospice deaths in DS cases between 1999 and 2020. In univariate logistic regression, using patients under one year as the reference group, those in the 45-54 age group were 8.817 times (OR=8.817, CI=7.079-10.98) more likely to die at home or hospice, followed by the 25-34 age group (OR=8.343, CI=6.431-10.825). Conversely, patients over 85 years were less likely to die at home or hospice (OR=0, CI=0-2.097e+69). In the gender groups, with women as the reference, men were 0.895 times less likely to die at home or hospice (OR=0.895, CI=0.844-0.95). In the census region group, using Region 1 (Northeast) as the reference, patients in Region 3 (South) were more likely to die at home or in hospice (OR=1.166, CI=1.071-1.269), whereas those in Region 4 (West) were less likely to die at home or in hospice (OR=0.973, CI=0.88-1.071). Regarding race, with Asian or Pacific Islanders as the reference group, American Indian or Alaska Native individuals were 2.389 times (OR=2.389, CI=1.465-3.895) more likely to die at home or hospice.

**Table 2:** Predictors of home or hospice death according to demographic variables The predictors of home and hospice death in Down syndrome cases from 1999 to 2020. *P-value <0.0.5 indicates significance. DS, Down syndrome

Variables	Univariate logistic regression		
	Odds ratio	95% confidence interval	P-value
Age			
<1 year	1.0 (Reference)		
1-4 years	3.673	(2.551, 5.29)	<0.001*
5-14 years	4.955	(3.343, 7.344)	<0.001*
15-24 years	7.536	(5.668, 10.018)	<0.001*
25-34 years	8.343	(6.431, 10.825)	<0.001*
35-44 years	8.023	(6.302, 10.212)	<0.001*
45-54 years	8.817	(7.079, 10.98)	<0.001*
55-64 years	7.911	(6.371, 9.824)	<0.001*
65-74 years	6.104	(4.836, 7.703)	<0.001*
75-84 years	5.095	(3.286, 7.899)	<0.001*
85+ years	0	(0, 2.097e+69)	0.903
Gender			
Male	0.895	(0.844, 0.95)	<0.001*
Female	1.0 (Reference)		
Census region			
Census region 1: Northeast	1.000 (Reference)		
Census region 2: Midwest	1.01	(0.923, 1.105)	0.831
Census region 3: South	1.166	(1.071, 1.269)	<0.001*
Census region 4: West	0.973	(0.884, 1.071)	0.578
Race			
American Indian or Alaska Native	2.389	(1.465, 3.895)	<0.001*
Black or African American	1.602	(1.203, 2.134)	0.001*
White	1.741	(1.326, 2.285)	<0.001*
Asian or Pacific Islander	1.000 (Reference)		

**Figure 1:**
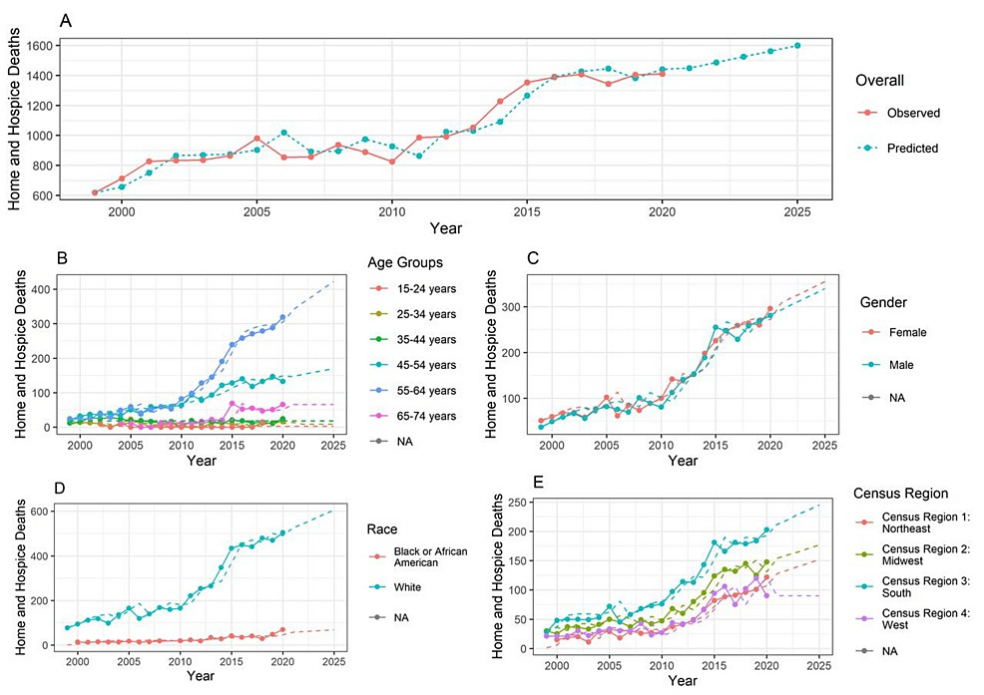
Mortality trends according to demographic variables The forecasting is done from year 1999 to 2025, with the training data available from year 1999 to 2020. In the line chart, the lines represent the observed data. The dotted line represents the forecasted data. (A) Overall trends; (B) trends according to age groups; (C) trends according to gender; (D) trends according to race; (E) trends according to census region of the United States.

Figure 1A shows the overall number of deaths in homes or hospitals. The graph indicates a gradually increasing trend with a notable upward surge after 2020. Between 1999 and 2010, the reported deaths consistently stayed below 1100. Over the next decade, with a steady increase, the number is predicted to reach 1400 cumulative reported deaths and is expected to surpass 1600 by 2025.

Figure 1B illustrates home and hospice deaths among different age groups. The graph indicates that patients in the 55-64 age group were more likely to die at home and hospice, showing a noticeable escalation after 2010 for this age group. In addition, the graph showed a general upward trend for the 45-54 and 65-74 age groups. No substantial changes in the reported deaths were observed for the other age groups between 1999 and 2020, for which a steady trajectory is expected over the next five years.

Figure 1C displays home and hospice deaths based on gender. The graph suggests that women are more likely to die at home and hospice centers than men, with a gradual and upward movement in the line charts after 2010.

Figure 1D depicts the number of home and hospice deaths among DS patients by race between 1999 and 2020, with predictions for the next five years. The graph shows that deaths among White patients exceeded those of Black or African American patients, surpassing 400 in 2015, and are predicted to exceed 600 by 2025.

Figure 1E highlights the gradually increasing trend in the line chart for the census region groups. The graph indicates a consistent rise in the number of reported deaths in home or hospice centers across all census regions, which is expected to continue with a similar upward trend. However, census region 4 (West) is presumed to maintain a linear trajectory between 2020 and 2025.

## Discussion

To evaluate and elucidate the mortality trends of individuals with DS, we analyzed data spanning 22 years (1999-2020) from the CDC-WONDER database. The data included a total of 6106 home or hospice care patients, 15302 patients in medical facilities and nursing homes, and 1196 patients in other facilities. DS is a chromosomal anomaly that results in poor survival, especially when associated with congenital heart defects (CHDs) [14].

The study highlighted several substantial findings. The highest number of deaths occurred in the 55-64-year age group, whereas the lowest number of deaths was in the 85+ year age group. Additionally, the 45-54 years age group closely followed in terms of the number of recorded deaths, with the majority occurring in medical facilities or nursing homes. The under-one-year age group also had a high number of deaths in medical facilities.

In contrast, a study by Motegi et al. observed the highest number of deaths among individuals with DS in the 20-year age group, with deaths increasing from 21.7% in 1995 to 69.9% in 2016 [15]. This discrepancy may be attributed to geographic, environmental, and genetic differences within the population sample in Motegi et al.’s study, which was based in Japan. Wright et al. found that CHDs adversely affected childhood survival outcomes in DS. Specifically, atrial septal defects were associated with early deaths (less than one year) and late deaths (greater than five years), whereas ventricular septal defects were associated with intermediate deaths (between one and five years) [14]. Thus, deaths in younger age groups of individuals with DS can be associated with comorbid conditions, such as CHD.

The survival of individuals with DS in the higher age group (55-64 years) in the United States can be attributed to a higher quality of medical care compared with other countries [16].

In terms of gender, men were observed to have the highest overall number of deaths, with the majority occurring in medical facilities or nursing homes. Conversely, the number of female deaths was higher than that of men in homes, hospices, and other facilities. This disparity could be attributed to female patients preferring home or hospice care over medical facilities or nursing home care [17].

These findings are consistent with a study by Motegi et al. on secular trends in longevity among people with DS in Japan between 1995 and 2016, which showed a higher number of deaths in women due to increased risk factors such as CHDs and the lack of equal availability of healthcare facilities for women [15]. Similarly, a study by Landes et al. found that women had an increased risk of mortality linked to factors such as CHD and Alzheimer’s disease, which can negatively affect life expectancy in individuals with DS [10].

In the present study, the White race had the highest number of deaths in all categories of place of death. The lowest number of deaths was recorded for American Indians or Alaska Natives; this difference may be due to the underreporting of causes of death in other races compared with White individuals with DS. In contrast, Santoro SL et al. observed that Black children with DS tend to have a higher mortality rate (8.9%) compared with White children (2.5%). The higher prevalence of congenital heart diseases among Black children under five years was found to contribute to this shorter life expectancy [18]. Similarly, a study by Day et al. in California showed that Black individuals with DS were at a greater risk for mortality than other races, with a relative risk of 1.5 [19]. This finding was supported by a study by Kucik et al., which found that the survival rate of Black children with DS was lower than that of White children with DS due to the presence of risk factors such as CHDs [20].

Based on the census data, region 3 (South) had the highest number of deaths in all categories, whereas the lowest number of deaths was recorded in region 1 (Northeast). This result may be due to the demographics of region 3, which has a higher number of Black individuals who are more vulnerable, especially in the under-five-year age group due to disparities in access to healthcare compared with the Northeast [21]. Overall, the highest number of deaths in each region in this study occurred in medical facilities or nursing homes, indicating a preference for these settings in end-of-life care.

Limitations

The study had several limitations. First, the latest data between 2021 and 2023 were not included in the analysis. This study could have greatly benefited from recent years’ data due to the dynamic nature of mortality trends, especially considering the impact of the COVID-19 pandemic. Including this data would have provided insights into healthcare preferences, availability, and the effects of COVID-19 on individuals with DS and their places of death. Additionally, post-pandemic trends in mortality for individuals with DS would have offered valuable information. Furthermore, the data in the study were not classified based on subcategories of DS, such as Trisomy 21 (nondisjunction), translocation DS, and mosaic DS [15].

## Conclusions

Patients with DS have experienced an increased number of deaths in home and hospice care over the past 10 years (1999-2020). Further analysis revealed that these deaths were more prevalent among individuals aged 55-64 years with DS. Women had a higher mortality rate in home and hospice settings compared with men. Between 1999 and 2020, White patients were found to be more affected by home and hospice deaths, with the South region also being substantially impacted.

These extensive analyses help us understand changing trends. For example, patients with DS often face various medical problems such as hearing impairment, respiratory infections, sleep apnea, congenital septal defects, and cognitive impairments. These conditions require different drugs and dosing, which can result in polypharmacy. The adverse effects caused by multiple medications can be numerous and potentially fatal. Therefore, determining when and which drugs to discontinue plays a crucial role in providing care to these patients. Additionally, many patients face psychosocial deficits, including social stigma, depression, and anxiety. Families also encounter numerous challenges, making it difficult to adapt and leaving them feeling unwelcome and distressed. A holistic approach is, therefore, required to prevent these deaths and address the various challenges faced by these patients and their families.
